# Sleep Parameters, Functional Status, and Time Post-Stroke are Associated with Offline Motor Skill Learning in People with Chronic Stroke

**DOI:** 10.3389/fneur.2015.00225

**Published:** 2015-10-30

**Authors:** Catherine Siengsukon, Mayis Al-Dughmi, Alham Al-Sharman, Suzanne Stevens

**Affiliations:** ^1^Department of Physical Therapy and Rehabilitation Science, University of Kansas Medical Center, Kansas City, KS, USA; ^2^Department of Rehabilitation Sciences, Jordan University of Science and Technology, Irbid, Jordan; ^3^Neurology Department, University of Kansas Medical Center, Kansas City, KS, USA

**Keywords:** sleep, chronic stroke, motor learning, offline, functional status

## Abstract

**Background:**

Mounting evidence demonstrates that individuals with stroke benefit from sleep to enhance learning of a motor task. While stage NREM2 sleep and REM sleep have been associated with offline motor skill learning in neurologically intact individuals, it remains unknown which sleep parameters or specific sleep stages are associated with offline motor skill learning in individuals with stroke.

**Methods:**

Twenty individuals with chronic stroke (>6 months following stroke) and 10 control participants slept for three consecutive nights in a sleep laboratory with polysomnography. Participants practiced a tracking task the morning before the third night and underwent a retention test the morning following the third night. Offline learning on the tracking task was assessed. Pearson’s correlations assessed for associations between the magnitude of offline learning and sleep variables, age, upper-extremity motor function, stroke severity, depression, and time since stroke occurrence.

**Results:**

Individuals with stroke performed with significantly less error on the tracking task following a night of sleep (*p* = 0.006) while the control participants did not (*p* = 0.816). Increased sleep efficiency (*r* = −0.285), less time spent in stage NREM3 sleep (*r* = 0.260), and more time spent in stage REM sleep (*r* = −0.266) were weakly-to-moderately associated with increased magnitude of offline motor learning. Furthermore, higher upper-extremity motor function (*r* = −0.400), lower stroke severity (*r* = 0.360), and less time since stroke occurrence (*r* = 0.311) were moderately associated with increased magnitude of offline motor learning.

**Conclusion:**

This study is the first study to provide insight into which sleep stages and individual characteristics may be associated with offline learning in people with stroke. Further research is needed to delineate which factors or combination of factors promote offline motor learning in people with neurologic injury to best promote motor recovery in these individuals.

## Introduction

Approximately 795,000 individuals in the United States experience a new or recurrent stroke each year (1) and more than half experience a persistent loss of function (2, 3). Furthermore, stroke is a leading cause of long-term disability in the United States (4). As rehabilitation following stroke often involves learning new as well as re-learning previously acquired motor skills, examining factors and mechanisms that impact motor learning and motor recovery must be explored.

Sleep may be a factor that could hasten motor recovery following stroke by enhancing motor learning (5, 6). Sleep has been shown to enhance motor learning and memory consolidation in young (7–10), middle-aged (11), and older adults (12). Recent work has demonstrated that sleep promotes learning of a functional walking task in young (13) as well as middle-aged and older adults (14). Participants who practiced a novel walking task and slept prior to the retest session walked faster around the path and demonstrated improved gait parameters while those who stayed awake between practice and retest did not demonstrate improvements in learning this task (13, 14). Complex tasks, such as walking, and perhaps functional tasks that are often practiced during rehabilitation may particularly benefit from sleep to promote learning.

Debate remains regarding which particular sleep stage or stages drive motor learning. Stage 2 sleep (15–18), and in particular sleep spindles (18–20), as well as stage REM sleep (21–23) has been associated with the learning of simple motor tasks. It has also been posited that rather than a particular sleep stage driving learning, it is the ordered sequence of non-REM sleep followed by stage REM sleep that promotes memory consolidation (24). Furthermore, stage REM sleep has been demonstrated to promote learning of more cognitively involved tasks (18, 23) and novel motor tasks (25) whereas stage NREM2 promotes learning of less cognitively involved motor tasks (18, 20) and tasks that the individual has some previous skill performing (25).

Studies demonstrate that individuals with chronic stroke benefit from sleep to enhance motor learning of a computer-based tracking task (5, 6). Individuals with chronic stroke who slept following practice of a continuous tracking task performed with less error at a retest session while those who stayed awake did not (5, 6). However, these initial studies were not conducted in a sleep laboratory so it is unclear if a particular sleep parameter or sleep stage is associated with sleep-dependent motor skill learning in individuals with chronic stroke.

Sleep disruptions are very common following stroke, occurring in up to 70% of individuals in the acute stage (26). Sleep issues following stroke include insomnia, excessive daytime sleepiness, obstructive sleep apnea, and restless leg syndrome. A reduction in total sleep time and sleep efficiency and an increase in waking after sleep onset are commonly reported (27–29). Furthermore, changes in sleep architecture frequently occur including a reduction in stage REM, stage NREM2, and stage NREM3 sleep and an increase in stage NREM1 (27, 30). Importantly, sleep issues do not appear to necessarily resolve with time; up to 50% of individuals with chronic stroke experience sleep dysfunction (31). However, the sleep parameters of individuals with chronic stroke are less well characterized than those with acute stroke. Studies indicate that individuals with chronic stroke may experience an increase in time spent in stage NREM2 sleep (31), an increase in sleep spindle activity (29), and a decrease in stage NREM3 sleep (32, 33). If stage NREM2 sleep (15), and in particular sleep spindles (18–20), is associated with sleep-dependent offline learning, perhaps an increase in stage NREM2 and sleep spindles in individuals with chronic stroke permits these individuals to benefit from sleep to enhance motor skill learning and potentially motor recovery.

The purpose of this study was to determine whether particular sleep parameters or specific sleep stages are associated with sleep-dependent offline motor skill learning in individuals with chronic stroke. Understanding how sleep promotes motor skill learning could lead to the manipulation of sleep parameters through use of medication or other means, an emphasis on the identification and treatment of sleep issues in people with stroke, as well as the strategic incorporation of sleep into rehabilitation sessions.

## Materials and Methods

Twenty-six individuals with chronic stroke (>6 months following stroke) and 10 neurologically intact adults were enrolled in the study. Participants were recruited from a stroke registry at the University of Kansas Medical Center, the local chapter of the American Stroke Foundation, area stroke support groups, and personal referral from physicians, participants, or other study personnel. Participants were included in the study if they: (1) were 40–75 years old, (2) had no known untreated sleep disorders, (3) maintained a regular sleep schedule (averaging 6–9 h of sleep per night on a sleep log maintained for a week prior to testing), and (4) score >26 on the Mini-Mental State Exam to insure cognitive abilities to complete the consent form. Individuals with stroke were included in the study if they had a unilateral stroke in the middle cerebral artery distribution confirmed either by magnetic resonance imaging or clinical presentation and were >6 months post-stroke. Participants were excluded from participating if they: (1) had an acute medical problem that prevented them from participating, (2) had uncorrected vision loss, (3) had history of admission for psychiatric issues, (4) had a history of more than one stroke, transient ischemic attacks, or extensive white matter disease, (5) identified themselves as a current smoker, and (6) had known uncontrolled depression. The study was conducted according to the regulations and with approval from the Institutional Review Board at the University of Kansas Medical Center. Informed consent was received from all participants.

Participants slept for three consecutive nights (see Table 1 for study design) in the Sleep Medicine Clinic sleep laboratory with polysomnography (PSG) using standardized techniques (34). The first night served as an acclimation night to allow participants to become accustomed to the sleep laboratory. The acclimation night also allowed for the detection of any unreported sleep disorders in the participants. Following the acclimation night, six participants with stroke were excluded from continuing in the study due to having an Apnea Hypopnea Index (AHI) ≥15. The second night of PSG was used as a baseline measure of sleep characteristics and sleep stages. The morning following the baseline PSG night, participants practiced a continuous tracking task and underwent retention testing of the task 24 h later. The participants underwent PSG recording the night between practice and retention testing (experimental night). The participants underwent a battery of cognitive tests following the retention test, but the cognitive data are not reported here. The PSG outcome variables of interest were total sleep time, sleep efficiency, and time spent in stages NREM1, NREM2, NREM3, and REM of the experimental night.

**Table 1:** **Study design**.

Day 1	Night 1	Day 2	Night 2	Day 3	Night 3	Day 4
	Acclimation		Baseline recording	Task practice (8:00 a.m. ± 1 h)	Experimental recording	Task retention test (8:00 a.m. ± 1 h)

The continuous tracking task has been previously used to demonstrate sleep-dependent motor skill learning in individuals with chronic stroke (5, 6). In brief, participants with stroke moved a joystick with their ipsilesional hand, which controlled a cursor on a computer screen. The less-affected, ipsilesional hand was used to assess motor learning while reducing the confound of motor execution impairments (35). Participants were instructed to try to match the cursor with the movement of the target. Control participants were matched for hand use. Participants practiced the continuous tracking task for 10 blocks, each block consisting of 10 trials for a total of 100 trials of practice. Each trial consisted of a repeated sequence segment embedded between two random segments. Participants did not receive explicit instruction on the presence of the repeating segment. The retention test consisted of 1 block (10 trials). The root mean square error (RMSE) for each trial was calculated, and the median RMSE was calculated for each block (6, 35).

In addition to the sleep data gathered using PSG, sleep quality was assessed using the Pittsburgh Sleep Quality Index (PSQI) (36), and current level of sleepiness was assessed at the practice and retention session using the Stanford Sleepiness Scale (37). Participants were asked to maintain a sleep log for a week prior to testing. Depression was assessed using the Geriatric Depression Scale (GDS) (38). Upper-extremity motor function was gathered from the individuals with stroke using the Fugl–Meyer Assessment of Physical Performance (FMUE) (39), and the Orpington Prognostic score (40) was used to assess stroke severity (Table 2).

**Table 2:** **Descriptive information**.

	Sex	Age	SSS1	SSS2	PSQI	GDS	Average sleep	Lesion side	Time post-stroke (month)	FMUE	Orpington
Stroke	7 M	59.9 (11.3)	1.7 (0.75)	1.8 (1.2)	4.8 (3.9)	4.4 (4.5)	7.5 (4.5)	7 L	76.8 (54.6)	55.9 (13.0)	1.9 (0.40)
	13 F							13 R
Control	4 M	61.3 (11.7)	1.7 (1.1)	1.7 (1.1)	4.0 (2.3)	3.1 (4.2)	7.5 (0.66)	–	–	–	–
	6 F	
*p*-value		0.747	0.882	0.829	0.664	0.525	0.989				

*Data are mean (SD). M, male; F, female; R, right; SSS1, Stanford Sleepiness Scale at practice session; SSS2, Stanford Sleepiness Scale at retention testing; PSQI, Pittsburgh Sleep Quality Index; GDS, Geriatric Depression Scale; Average sleep, average amount of sleep the week prior to testing determined by sleep log; FMUE, Upper-extremity motor portion of Fugl–Meyer*.

Group differences in age, sleep quality, depression, average sleep for a week prior to testing, and sleepiness at practice and retention testing were assessed using one-way ANOVAs. A repeated measures ANOVA model was used to generate parameter estimates to assess for change in performance on the motor learning task from the last practice block to the retention block. An offline learning score was calculated by subtracting the last practice block RMSE from the retention block RMSE so a more negative offline learning score indicates a higher magnitude of change in performance. Exploratory Pearson’s correlations were used to assess for associations between the magnitude of offline learning for individuals with stroke and the PSG sleep variables of interest, sleep quality, age, upper-extremity motor function, stroke severity, depression, and time since stroke occurrence. *Post hoc* analysis using one-way ANOVAs was used to compare select demographic characteristics of those individuals with stroke who demonstrated offline motor learning (“learners,” *n* = 14) with those individuals with stroke who did not demonstrate offline motor learning (“non-learners,” *n* = 6).

## Results

**Figure 1:**
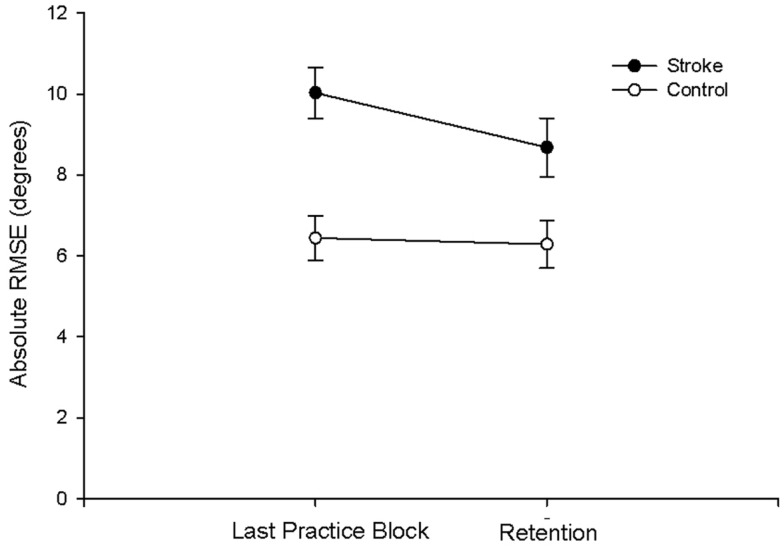
**Absolute RMSE performance at last practice block and at retention for the stroke and control group**. Individuals with stroke demonstrated a significant improvement in tracking performance from the last practice block to the retention block (*p* = 0.006); the control participants did not demonstrate a significant improvement (*p* = 0.816).

**Figure 2:**
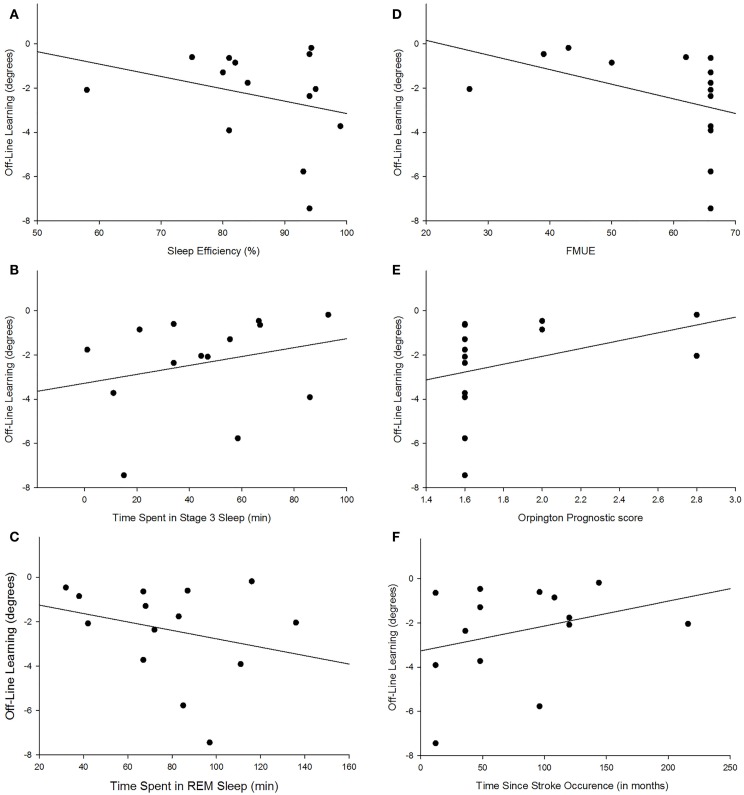
**Scatterplot depiction of association between magnitude of offline learning and sleep efficiency [*r* = −0.285; (A)], time spent in NREM3 [*r* = 0.260; (B)], time spent in REM [*r* = −0.266; (C)], upper-extremity motor function [*r* = −0.400; (D)], lower stroke severity [*r* = 0.360; (E)], and less time since stroke occurrence [*r* = 0.311; (F)]**.

There were no group differences between the participants with stroke and the control group in age (*p* = 0.747), sleep quality (*p* = 0.664), depression (*p* = 0.525), average sleep for a week prior to testing (*p* = 0.989), sleepiness at practice (*p* = 0.882), or sleepiness at retention testing (*p* = 0.829; Table 2). Individuals with stroke demonstrated a significant improvement in tracking performance from the last practice block to the retention block (*p* = 0.006) while the control participants did not (*p* = 0.816; Figure 1). While none of the correlations were statistically significant, correlations with *r* > 0.25 are reported. For individuals with stroke who demonstrated offline motor learning of the tracking task, increased sleep efficiency (*r* = −0.285; Figure 2A), less time spent in stage NREM3 sleep (*r* = 0.260; Figure 2B), and more time spent in stage REM sleep (*r* = −0.266; Figure 2C) were minimally associated with increased magnitude of offline motor learning. Furthermore, higher upper-extremity motor function (*r* = −0.400; Figure 2D), lower stroke severity (*r* = 0.360; Figure 2E), and less time since stroke occurrence (*r* = 0.311; Figure 2F) were moderately associated with increased magnitude of offline motor learning. There were no differences between the stroke learners and non-learners for age (*p* = 0.716), sleep quality (*p* = 0.116), depression (*p* = 0.352), average sleep for a week prior to testing (*p* = 0.726), sleepiness at practice (*p* = 0.818), or sleepiness at retention testing (*p* = 0.213).

## Discussion

This study confirms the results of prior studies (5, 6) that individuals with stroke benefit from sleep to promote motor skill learning while age- and gender-matched controls do not. The mounting evidence that motor skill learning in individuals with stroke is enhanced by sleep has very important clinical implications, including the need to emphasize screening individuals with stroke for sleep issues, determining which factors or combination of factors (i.e., sleep stage combined with individual characteristics) drive offline motor learning, and how to manipulate or influence those factors to enhance motor learning and potentially recovery following stroke.

In this study, 6 of 26 individuals (23%) with chronic stroke were excluded due to having an AHI ≥15 and were referred to a sleep neurologist for further assessment and possible treatment. It is disconcerting that nearly a quarter of the participants with stroke were unaware that they had at least moderate sleep apnea as sleep apnea has been associated with increased risk of future strokes (41). Evidence suggests up to 50% of individuals with chronic stroke experience sleep issues (31). Thus, physicians may need to consider screening and testing individuals following stroke for possible sleep disturbances.

The mounting evidence that sleep enhances motor learning in individuals with stroke demonstrates the critical need to understand which sleep parameters or individual characteristics are associated with sleep-dependent motor learning. The finding of this study that increased sleep efficiency is weak-to-moderately associated with a higher magnitude of sleep-dependent offline motor learning is not surprising. Sleep efficiency which is the percent of total time in bed spent sleeping is generally considered a measure of sleep quality. Therefore, it is not the total amount of sleep but the quality of sleep that is associated with motor skill learning.

The finding of this study that more time spent in stage REM sleep is associated with a higher magnitude of offline motor learning is supported by prior studies (21, 23, 25). The continuous tracking task used in this study requires participants to implicitly learn the upper-extremity movements associated with moving a joystick to track a repeating sequence. Furthermore, this movement and tracking pattern is a novel task for all participants. Tasks that require learning a rule and are cognitively more involved have been associated with stage REM sleep (18, 23) as have tasks that are novel to the individual (25). Several cortical regions that are activated during the execution of motor tasks including the supplementary motor cortex, premotor cortex, and primary sensory motor cortex are more active during REM sleep, suggesting that memories are consolidated during REM sleep (21, 42, 43).

The finding that less time spent in stage NREM3 is associated with higher offline motor learning score appears contrary to prior studies (33, 44). Huber et al. (44) found that an increase in slow wave activity was associated with better performance on learning a rotational task in neurologically intact young adults. A recent study by Poryazova et al. (33) suggests that the increase in slow wave activity during sleep is a sign of neuronal plasticity in the brain and hence might be associated with increased motor learning.

While the positive association with sleep efficiency and REM sleep and the negative association with stage NREM3 sleep suggest that these sleep parameters contribute to offline motor learning, they are likely not driving offline motor learning in individuals with chronic stroke due to the weak-to-moderate association. The strength of association may be due to the small sample size. Also, it may be that the individual’s characteristics may interact with the sleep stages or act in conjunction with the sleep stages to promote offline motor learning. The findings from this study that higher upper-extremity motor function, lower stroke severity, and less time since stroke occurrence were moderately associated with increased magnitude of offline motor learning suggest that the individual’s characteristics likely impact sleep-dependent skill learning. However, which specific characteristics and how those characteristics interact with the sleep stages to promote motor learning in individuals with stroke remains to be determined. A future larger scale study with adequate sample size is needed to assess these interactions.

The findings support prior studies that healthy older adults fail to demonstrate sleep-dependent offline motor skills learning on simple computer-based tasks (6, 11, 45). The lack of offline improvement in tracking performance by the healthy older control participants is likely not due to a ceiling effect. The most accurate tracking performance during practice was 4.16° by a control participant and 4.37° for a participant with stroke. Therefore, the participants had the potential to perform the task with this degree of accuracy at retention, indicating that a ceiling effect is an unlikely explanation for why the control participants failed to benefit from sleep to enhance learning of a motor task.

Future studies are needed to investigate how individual’s characteristics may interact with sleep or impact the ability of sleep to enhance motor learning following stroke. Furthermore, research is needed to consider how sleep may be manipulated or facilitated to promote motor learning and potentially hasten motor recovery following stroke. In addition, clinicians may consider screening for sleep disorders in people with chronic stroke despite lack of subjective complaints.

## Conflict of Interest Statement

The authors declare that the research was conducted in the absence of any commercial or financial relationships that could be construed as a potential conflict of interest.
